# Key mechanisms governing resolution of lung inflammation

**DOI:** 10.1007/s00281-016-0560-6

**Published:** 2016-04-27

**Authors:** C. T. Robb, K. H. Regan, D. A. Dorward, A. G. Rossi

**Affiliations:** MRC Centre for Inflammation Research, The Queen’s Medical Research Institute, University of Edinburgh Medical School, 47 Little France Crescent, Edinburgh, EH16 4TJ UK

**Keywords:** Neutrophils, Eosinophils, Macrophages, Lung inflammation, Lung diseases, Pro-resolution mediators, Apoptosis, ETosis, Efferocytosis

## Abstract

Innate immunity normally provides excellent defence against invading microorganisms. Acute inflammation is a form of innate immune defence and represents one of the primary responses to injury, infection and irritation, largely mediated by granulocyte effector cells such as neutrophils and eosinophils. Failure to remove an inflammatory stimulus (often resulting in failed resolution of inflammation) can lead to chronic inflammation resulting in tissue injury caused by high numbers of infiltrating activated granulocytes. Successful resolution of inflammation is dependent upon the removal of these cells. Under normal physiological conditions, apoptosis (programmed cell death) precedes phagocytic recognition and clearance of these cells by, for example, macrophages, dendritic and epithelial cells (a process known as efferocytosis). Inflammation contributes to immune defence within the respiratory mucosa (responsible for gas exchange) because lung epithelia are continuously exposed to a multiplicity of airborne pathogens, allergens and foreign particles. Failure to resolve inflammation within the respiratory mucosa is a major contributor of numerous lung diseases. This review will summarise the major mechanisms regulating lung inflammation, including key cellular interplays such as apoptotic cell clearance by alveolar macrophages and macrophage/neutrophil/epithelial cell interactions. The different acute and chronic inflammatory disease states caused by dysregulated/impaired resolution of lung inflammation will be discussed. Furthermore, the resolution of lung inflammation during neutrophil/eosinophil-dominant lung injury or enhanced resolution driven via pharmacological manipulation will also be considered.

## Introduction

Acute inflammatory responses are initiated by injury, infection and irritation which, in turn, protect the host from systemic infection and help to restore tissue homeostasis [1]. Inflammation therefore represents a crucial defence mechanism that is protective and vital to health [2, 3]. Typically, the molecular events and cellular interplays prevalent during acute inflammatory responses are efficient at minimising impending injury, infection or irritation, which leads importantly to restoration of tissue homeostasis and thus complete resolution of the acute inflammatory response. However, if an acute inflammatory response is mounted that is uncontrolled in terms of magnitude or duration, it can lead to disease [1, 3]. In the lung, dysregulated acute inflammation can result in lung injury contributing to pulmonary fibrosis that severely impairs essential gas exchange processes. Therefore, numerous mechanisms exist, which tightly regulate the gravity and duration of lung inflammation. If unresolved, acute lung injury (ALI) and/or lung inflammation can progress to chronic inflammation, which occurs in lung diseases such as acute respiratory distress syndrome (ARDS), asthma, cystic fibrosis (CF) and chronic obstructive pulmonary disease (COPD) [1].

Pro-resolution of inflammation was previously regarded as a passive process, with limited understanding of mechanisms regulating the resolution of inflammation. However, over the years, substantial research in this field has identified inflammation resolution as an active and highly regulated cellular and biochemical process. It is now known that numerous molecular mediators of inflammation exist, including many pro- and anti-inflammatory cytokines and chemokines, with attenuation of pro-inflammatory mediator effects assisting in the successful ‘switching off’ of inflammation [4]. More recently, several endogenous pro-resolving bioactive lipid mediators (immunoresolvents) have been discovered such as lipoxins, resolvins, protectins and maresins, which are heavily involved in driving ‘programmed resolution’ that successfully terminate inflammation [5–8]. Other key processes governing the successful resolution of inflammation include the phagocytic clearance of apoptotic cells [9, 10] during a process referred to as efferocytosis that also results in the phagocytic cells, switching phenotype from a pro-inflammatory cell to a more anti-inflammatory/pro-resolution phenotype [10, 11]. Also, pertinent to the lung mucociliary clearance of infective agents, allergens, foreign particles and effete cells occur [12]. This review encompasses the cellular mechanisms and chief biochemical mediators involved in the resolution of lung inflammation and repair of damaged tissues, with a specific focus on neutrophil/eosinophil-dominant lung inflammation and pharmacological approaches to drive resolution [13–16].

### Cells of the innate immune system

Antigen-independent innate immunity provides the first line of leukocytic defence against invading microorganisms during which inflammation is an early key response to infection, injury or irritation. Innate immune defence during lung inflammation involves several cell types and cellular interplay. These include leukocytes such as polymorphonuclear granulocytes (neutrophils, eosinophils, basophils) and agranulocytes (monocytes, macrophages), lung epithelial/endothelial cells, mast cells, natural killer (NK cells) and dendritic cells. These cells can influence the function of other cell types, such as innate lymphoid cells [17] and lymphocytes [18], which are not specifically covered in this current review.

#### Neutrophils

Neutrophils, accounting for 70 % of the circulating human blood leukocytes, are short lived in the circulation surviving for up to 7–10 h (although the precise length of time in the circulation remains controversial; see Tak et al [19]). However, during an inflammatory scenario or in response to chemical stimuli, they can survive up to, or greater than, 48 h. These cells, typically 12–15 μm in diameter, contain a distinct multi-lobed nucleus and possess four different types of granules, primary (azurophilic), secondary (specific), gelatinase and secretory. These granules contain >300 proteins which are involved in several cell processes including migration, adhesion and anti-microbial activity [20]. Neutrophils are very versatile and upon inflammatory insult, rapidly migrate to the foci of injury/infection, where they are often first on the scene and help defend the host via phagocytosis, degranulation, generation of reactive oxygen species (ROS) or by releasing webs of chromatin via neutrophil extracellular traps (NETs) generation to trap and kill microorganisms. Furthermore, there is evidence that neutrophils can change from a pro-inflammatory to an anti-inflammatory phenotype following a successful inflammatory response. In such an instance, neutrophils stop producing and releasing pro-inflammatory mediators such as leukotriene B_4_ (LTB_4_) and platelet-activating factor (PAF) and start to produce and release pro-resolving mediators such as pro-resolving bioactive lipids (e.g. lipoxins) which enhance the resolution phase of inflammation [21], for a thorough review of the pro-resolution properties exhibited by neutrophils [see 22]. Once the neutrophil has fulfilled its physiological function, they normally undergo apoptosis which retains membrane integrity, thus preventing uncontrolled release of noxious cellular contents and internalised microbes to the immediate extracellular environment [23]. Apoptosis-specific cell changes promote the recognition and uptake of neutrophils by phagocytes such as macrophages, dendritic cells and epithelial cells. It is essential for the successful resolution of inflammation that neutrophils are ‘switched off’, undergo apoptosis and are successfully cleared. If unresolved, acute inflammation can lead to chronic inflammation where mass neutrophil influx to a localised vicinity results in host tissue damage. This can occur when excess neutrophils produce free radical species during ROS generation, release proteolytic and anti-microbial granule contents during de-granulation and externalisation chromatin studded with nuclear, granular and cytosolic proteins with high anti-microbial properties during NET generation. Moreover, during hypoxic conditions (1 % oxygen) which are commonly experienced throughout inflammation, hypoxia-induced neutrophil survival is observed, mediated by hypoxia-inducible factor (HIF)-1α-dependent nuclear factor kappa light chain enhancer of activated B cells (NF-ƘB) activity and prolyl hydroxylase 3 expression. Like lymphocytes and macrophages, evidence suggests that neutrophils can also exist as a heterogeneous population exhibiting different phenotypes [24, 25]. Interestingly, accumulating recent evidence indicates that neutrophils, depending of the inflammatory response, may be capable of leaving the vicinity of inflammatory site by a process termed reverse migration [26–32]. Neutrophil-dominant inflammation is implicated in a number of inflammatory lung diseases including ALI/ARDS, CF, COPD, idiopathic pulmonary fibrosis (IPF), bronchiectasis, atopic/non-atopic asthma and severe asthma, during which neutrophil numbers are elevated (neutrophilic asthma).

#### Eosinophils

These cells make up <5 % of the circulating human blood leukocytes and can survive for up to 12 h but if necessary have the ability to prolong their life span for at least a week. They are slightly larger than neutrophils with a diameter of 12–17 μm, possess a bi-lobed nucleus and are easily identifiable via Romanowsky staining (methylene blue and eosin). Like neutrophils, eosinophils are loaded with granules in their cytoplasm; however, eosinophilic granules contain different components such as eosinophilic cationic protein, major basic protein, eosinophil peroxidase and eosinophil-derived neurotoxin, which are cytotoxic to airway epithelial cells. On arrival to the site of injury/infection (especially parasitic infection), eosinophils undergo degranulation which aids in removal of the inflammatory stimuli circumventing further inflammation. Eosinophils can also contribute to host defence via release of eosinophil extracellular traps (EETs) composed of either mitochondrial or nuclear DNA [33, 34]. These EETs contribute to anti-microbial defence via release of mitochondrial DNA that associates with eosinophil-derived granule proteins capable of capturing and killing microorganisms in vitro and in vivo [33]. Eosinophils can also undergo apoptosis, which are then cleared via phagocytosis by macrophages, modulated by IL-5 [35]. Eosinophils are capable of displaying both pro-inflammatory and anti-inflammatory phenotype and function. Anti-inflammatory activities of eosinophils include an important regulatory role during hypersensitivity reactions via eosinophil peroxidase-mediated inactivation of LTB_4_, C_4_ and D_4_ [36]. However, eosinophil peroxidase can also exert pro-inflammatory activities in areas of inflammation where mast cells and eosinophils are both present, largely via extracellular formation of active complexes formed between eosinophil peroxidase and mast cell granules [37]. Anti-viral activity has also been documented for human eosinophils and their associated ribonucleases against respiratory syncytial virus (in vitro) and for mice eosinophils and associated ribonucleases against pneumonia virus of mice in vivo (for review, see Rosenberg and Domachowske [38]). Furthermore, human and mouse eosinophils are capable of displaying anti-viral activity against parainfluenza 1 in the lung (a common respiratory virus) [39]. Eosinophils are dominant during allergic airway inflammation [40], including atopic/non-atopic asthma and allergic rhinitis, and are known to be involved in the maintenance and restoration of lung homeostasis.

#### Basophils

Basophils represent the rarest of the circulating human granulocytes, and their granules contain a variety of substances including histamine, heparin, serotonin, neutral proteases and hydrolases. Upon stimulation, they are capable of releasing their granule contents and synthesis of mediators including bioactive lipids and cytokines. Thus, upon exposure to allergens, they become activated and rapidly degranulate, which exaggerates inflammation during atopic/non-atopic asthma and allergic rhinitis [41].

#### Monocytes/macrophages

Monocytes contain numerous granules smaller than those of their granulocyte counterparts, which mostly contain lysosomal enzymes which aid in the destruction of internalised phagocytosed microorganisms. In the absence of inflammation, monocytes are normally confined to the bone marrow and blood. However, upon inflammatory insult, they rapidly migrate to inflamed tissue and differentiate into large tissue resident phagocytic macrophages. Depending on the microenvironment, macrophages can change their status to a variety of phenotypes. Please note that for convenience, macrophages have been classified into different phenotypes; this nomenclature is not precise and the authors note that the macrophage is plastic and can change depending on environment, origin and activation status. For example, they can have a more pro-inflammatory phenotype (often termed as M1 or classical macrophages), anti-inflammatory phenotype (termed M2 or alternative macrophages) or pro-resolving phenotype [11, 42, 43]. M1 switching can be induced by intracellular pathogens, bacterial cell wall components such as lipopolysaccharide (LPS), lipoproteins and soluble mediators such as the cytokines interferon gamma (IFNγ) and tumour necrosis factor (TNF), which, in turn, lead to release of various pro-inflammatory cytokines/mediators (IL-1, IL-6, IL-8, TNF, IFNγ, LTB_4_) exacerbating inflammation, as well as nitric oxide (NO) generation which aids in efficient killing of microorganisms [44]. M2 switching can be induced by various parasites and fungal cells; immune complexes; apoptotic cells; and soluble mediators including macrophage colony-stimulating factor (M-CSF), IL-4, IL-10, IL-13 and transforming growth factor beta (TGFβ) [45]. M2 macrophages shut down the release of pro-inflammatory stimuli and release pro-repair and anti-inflammatory cytokines/mediators such as IL-10, TGFβ and prostaglandin E_2_ (PGE_2_). Furthermore, M2 macrophages have enhanced phagocytic capabilities, with their most important function being the efficient clearance of apoptotic cells [46], which contributes largely to the successful resolution of inflammation. During lung infection and injury, migration and retention of monocyte and macrophage populations are implicated in triggering and sustaining pulmonary inflammation [47].

#### Lung epithelial/endothelial cells

In the airways, the trachea, combined with main bronchi, constitute proximal cartilaginous airways and are responsible for the conduction of inhaled air. During breathing, the proximal pseudostratified epithelium participates in defence against environmental toxins and invading microorganisms. Conversely, a more columnar epithelium is located in distal airways, where high numbers of goblet and ciliated epithelial cells are situated. Goblet epithelial cells secret mucous, which provides lubrication to assist ciliated epithelial cells in sweeping microorganisms, dust particles and effete cells away from the lungs (mucociliary clearance). The microvascular pulmonary endothelium and epithelial lining of the alveoli form the foundation of the gas exchanging air-blood barriers in the lung. This barrier is composed of three distinct compartments, blood, interstitium and the alveolar space [48]. The alveolar epithelium is made up of type-1 and type-2 alveolar cells (pneumocytes), with gas exchange performed by type-1 cells and excretion of pulmonary surfactant performed by type-2 cells, which maintains normal lung function by reducing surface tension. Alveolar macrophages are also the most abundant phagocytes present in alveolar space within the lung. Owing to their large surface area and the constant onslaught from microorganisms and particulates present in the air, lungs have acquired effective mechanisms for the detection of microbes. In infants, developmental disorders in the extensive interface where alveolar endothelial cells are directly opposite alveolar epithelial cells can lead to severe respiratory complications. In mature lungs, irreparable changes to the structure of the blood-gas interface contribute to fibrotic lung diseases and pulmonary emphysema, whereas dysfunction in the lung endothelial/epithelial cell barrier is a major contributor of ALI. Moreover, in ALI/ARDS, extensive damage to the endothelial/epithelial cell barriers causes leakage of edema fluid and inflammatory cells into the alveolar spaces resulting in hypoxemia and respiratory failure. In terms of reparative capabilities, endothelial cells can facilitate epithelial repair in the lung microenvironment [49].

#### Mast cells, NK cells and dendritic cells

Mast cells are thought to be involved in wound healing and repair and are found in skin and mucosal/connective tissues, where upon response to a pathogenic insult preferentially concentrate within mucosal/connective tissues to provide innate immune defence. Mast cells (via degranulation) can release potent inflammatory mediators including histamines, proteases, chemotactic factors, cytokines and arachidonic acid metabolites that impact upon the vasculature, smooth muscle, connective tissue, mucous glands and other inflammatory cells [50]. Like eosinophils and basophils, mast cells are implicated in allergic airway inflammation, with mast cell numbers elevated in pulmonary alveoli and airways, as well as in asthmatic lungs or in bronchial alveolar lavage (BAL) fluid from patients with IPF and sarcoidosis [51]. Other cell types involved in lung inflammation include NK cells and dendritic cells. NK cells are cytotoxic lymphocytes and can play both advantageous and disadvantageous roles during asthma, COPD, influenza and tuberculosis. However, there remains a paucity of knowledge as to how the functions of these cells are regulated in the unique tissue environment of each condition [52]. Dendritic cells are the messengers between the innate and adaptive immune systems. In the lung, dendritic cells create a cellular interphase between the external environment and the microenvironment. Lung dendritic cells play significant roles during the pathogenesis of asthma via regulation of bronchial hyperreactivity, recruitment of eosinophils/mast cells to localities of airway inflammation and induction of hyperplasia in goblet cells [53].

### Cell death

Cell death processes are tightly regulated to safeguard successful resolution of inflammation. Nevertheless, dysregulation of cell death commonly occurs hampering the pro-resolution process. In the lung, numerous cell death processes govern inflammation. Understanding the mechanisms that regulate cell death in the lung will help enable identification of novel therapeutic targets to limit/resolve inflammation and restore homeostasis.

#### Apoptosis

Granulocyte apoptosis has been a subject of much interest over recent decades, and there is strong evidence that failure of inflammation resolution contributes to numerous chronic inflammatory conditions and with its manipulation, therefore offering potential novel therapeutic targets. Inflammatory cells have the potential to be incendiary in the host tissue environment and, in the absence of an appropriate inflammatory ‘threat’, can trigger host tissue damage secondary to release of histotoxic mediators such as proteases and reactive oxygen species. Perhaps one of the most critical mechanisms for resolution and restoration of tissue homeostasis following an acute inflammatory insult is the ability of accumulated migratory granulocytes to undergo immunologically silent programmed cell death, namely, apoptosis. This highly regulated, energy-dependent and complex process involves the coordinated destruction and packaging of inflammatory cell contents for phagocytic clearance in a manner that does not elicit a host immune response, facilitates healing, and promotes and maintains self-tolerance by the adaptive immune system to create immunological memory. In addition, this neat packaging of cell contents prevents the leakage of pro-inflammatory mediators and contains histotoxic weaponry, including proteases, reactive oxygen species production and lysozymes. Granulocyte apoptosis is a caspase-dependent process that proceeds following activation of one of two major pathways, the intrinsic and extrinsic [1, 54]. It has become increasingly evident that the mutual exclusivity of these pathways is not as clear-cut as was previously assumed, and there is a degree of cross talk between the molecular component of their execution, with both ultimately dependent on the actions of caspases to initiate cell suicide. Caspases, a family of cysteine-aspartic proteases, are the critical intracellular mediators of apoptosis and are also implicated in inflammation and necrosis, thus offering a promising target for pharmacological manipulation [55, 56].

The intrinsic, or mitochondrial, pathway occurs when the balance of pro- and anti-apoptotic mediators of the Bcl-2 family proteins tips in favour of cell death, which occurs in response to DNA damage or endoplasmic reticulum stress. In the mature granulocyte, the pro-apoptotic family members, Bax, Bad, Bak and Bid, are suppressed by their anti-apoptotic counterparts, Mcl-1, Bcl-xl and A1, thus maintaining cell viability. In the presence of sufficient cellular stress, they circumvent this suppression and translocate from cytoplasm to mitochondria, triggering development of mitochondrial outer membrane permeabilisation (MOMP). MOMP allows mitochondrial molecules cytochrome C, Smac/DIABLO, Omi/HtrA2 and serine proteases to enter the cytosol, where cytochrome C interacts with Apaf-1 to form the apoptosome, which is ultimately responsible for cell death via the activation of pro-caspase 9. The resultant caspase 9 causes cleavage of the ‘executioner’—caspase 3, leading to DNA fragmentation, cross-linking and degradation of intracellular proteins and membrane receptor switch. Conversely, apoptosis advancing via the extrinsic ‘death receptor’ pathway occurs in response to stimulation by extracellular mediators, primarily TNF, Fas ligand and tumour necrosis factor-alpha-related apoptosis-inducing ligand (TRAIL) [57]. These intercellular messengers activate receptors on granulocyte plasma membranes—specifically TRAIL receptor (TRAIL-R), TNF receptor 1 (TNFR1) and Fas receptor (FasR), which upon binding with their corresponding ligand are prompted to coalesce. Assemblages of membrane proteins bind with internal adaptors, forming death domain proteins that attract clusters of cytosolic pro-caspase 8. The interactions of these proteins trigger an intracellular cascade, namely, the death-inducing signalling complex (DISC) that culminates in autocatalytic cleavage of pro-caspase 8, which then results in apoptosis of the cell again via cleavage of caspase 3. Caspase 8 generated in response to extracellular Fas ligand is the main executor of cross talk between the intrinsic and extrinsic pathways, as its release triggers MOMP via cleavage of Bid [58, 59].

Following caspase activation, nuclear DNA forms nucleosomes, dense packages of genetic material. Occurring simultaneously is the alteration of the plasma membrane receptor profile. The pro-survival molecule suite, which includes CD47 and CD31, is replaced by a milieu of ‘find-me’ and ‘eat-me’ signals that trigger recognition and stimulate uptake of the dying cell by macrophages or other cells with phagocytic capacity [10, 60]. Find-me signals are released from apoptotic cells which subsequently attract nearby phagocytes. In mammals, several find-me signals have been identified including fractalkine (CX3CL1), lysophosphatidycholine (lipid mediator), sphingosine 1-phosphate and nucleotides including adenosine triphosphate and uridine 5′ triphosphate [61–64]. Eat-me signals allow the specific recognition of apoptotic cells via cell different cell surface changes which include exposure of phosphatidylserine (PS) to the outer membrane leaflet, intracellular adhesion moelecule-1 (ICAM1) epitope alteration, exposure of calreticulin and alteration of cell surface charge and glycosylation configurations (for review, see Gardai et al. [65]). The best described and most evolutionarily conserved of these eat-me signals is the externalisation of PS to the outer membrane leaflet [66, 67], which along with ICAM3 and annexin 1 promotes phagocytosis. Additionally, find-me signals such as nucleotides, fractalkine and lipid mediators attract not only professional phagocytes but can also facilitate uptake by neighbouring cells and other non-professional phagocytes including bronchial epithelial cells [10, 68, 69]. In addition to preventing direct, though inadvertent, damage to host tissues, this mechanism of removal dampens the immune response and encourages resolution, allowing normal tissue homeostasis to resume. Apoptosis is an important clearance mechanism for effete cells and for the successful resolution of lung inflammation. Granulocyte apoptosis has been shown to be delayed in lung disease, and specific induction of granulocyte apoptosis can enhance the resolution of lung inflammation (for in-depth reviews, please refer to [1, 14]).

#### Other cell death processes in the lung

In direct opposition to its well-tempered counterpart (apoptosis), necrosis results in loss of membrane integrity and the unrestrained release of intracellular contents following cell trauma. The release of toxic damage-associated molecular patterns (DAMPs) into the extracellular environment characteristically results in an acute inflammatory response with inflammatory cell influx, paracrine effects on surrounding cells with release of pro-inflammatory mediators and significant potential for host tissue destruction [70, 71]. A variety of insults, including infection, chemicals, physical trauma and nutritional deficits, cause direct loss of membrane integrity—so-called primary necrosis. In situations where there is a failure in the timely and sufficient clearance of apoptotic cells by phagocytes, secondary necrosis occurs due to the inevitable disintegration of the apoptotic cell membrane, which may result in a late-phase inflammatory response, the nature of which is still debated [10, 60, 68]. There is increasing recognition of the importance of DAMPs in the propagation of the acute inflammatory response within the lung through interaction with pathogen recognition receptors (PRRs) [72]. Their activation promotes inflammation via transcription of pro-inflammatory cytokines and enhances the anti-microbial response. There is growing evidence to suggest that secondary necrosis, the necrotic fate of a cell following a failure of phagocytosis once apoptosis has occurred, contributes to persistent inflammation in a number of chronic conditions, and certainly evident that necrosis in the context of hyperactive acute response can result in significant long-term sequelae as a result of tissue damage and aberrant remodelling.

In 2004, a novel cell death process distinctly separate to necrosis and apoptosis was discovered when it was observed that human neutrophils could generate NETs [73] for innate immune defence. NETs are composed of decondensed nuclear chromatin that is discharged to the extracellular environment in a controlled manner. Additionally, neutrophils can release mitochondrial DNA [74]; however, mitochondrial DNA is 100,000 times less abundant on NETs than nuclear DNA [75]. NETs are characterised by the nuclear membrane being entirely fragmented with most of the granules being dissolved, thus allowing direct contact and mixing of nuclear, cytoplasmic and granular components [76]. Studded on the DNA backbone of NETs are nuclear, granule and cytosolic proteins. Nuclear proteins include citrullinated histones and anti-microbial peptides (AMPs) such as the cathelicidin, LL37; azurophilic (primary) granule proteins such as neutrophil elastase (NE), cathespin G, myeloperoxidase (MPO) and α-defensins; specific proteins from secondary and tertiary granules such as lactoferrin and gelatinase, respectively; or cytosolic proteins such as the cytosolic protein complex, calprotectin [73, 77]. The core histones H2A, H2B, H3 and H4 account for 70 % of all NET-associated proteins [77]. Histone hypercitrullination which mediates chromatin decondensation during NET formation is mediated via peptidylarginine deiminase 4 (PAD4) [78].

The formation of NETs is dependent upon generation of ROS via activation of nicotinamide adenine dinucleotide phosphate (NADPH) oxidase, actin filament polymerisation, as well as requiring activation of protein kinase C (PKC) pathways upstream of NADPH oxidase [76, 79, 80]. NETs have the ability to capture and kill both Gram-positive/negative bacteria, viruses, fungi and larger parasites [73, 81–83]; however, it is now widely regarded that NETs are more efficient at trapping microorganisms as opposed to killing. Having said that, bacteria do have the ability to escape and degrade NETs via numerous mechanisms, for example, polysaccharide formation which causes electrochemical repulsion of AMPs or DNAse generation aiding degradation of chromatin [84, 85]. This process was first termed NETosis [86] as it was thought to be exclusive to neutrophils. However, this cell death pathway is now commonly referred to as ETosis [87] and can be found in a number of different immune cell types, as well as in haemocytes of lower invertebrates [88]. The early origin of ETosis helps explain some of its pathological effects in mammals where ETosis can be viewed as a double-edged sword.

ETosis is implicated in a number of chronic lung inflammatory disorders, including ALI and ARDS, influenza pneumonia, cystic fibrosis, asthma, COPD and tuberculosis. A hallmark of infection-related ALI/ARDS and in sterile injury is the activation and subsequent mass migration of neutrophils into the alveolar space, which is initiated by chemokines released from macrophages, neutrophils and epithelial cells [89]. Neutrophil activation and NET formation in the alveolar space are initiated by a highly localised concentration of stimulating factors. Injury to alveolar epithelial cells increases permeability of the barrier between the alveolar space and blood vessels, which also promotes the epithelium to release pro-inflammatory IL-8. This can result in leakage of edema fluid containing high infiltrating numbers of neutrophils into the alveolar space. Within the alveoli, NETs are released in response to host-derived factors such as granulocyte/macrophage colony-stimulating factor (GM-CSF), complement factor 5a (C5a), activated platelets and singlet oxygen. NETs then cause secondary epithelial cell damage via release of NET proteins and ROS generation, which results in chronic inflammation. Potent lung injury factors released by NETs include NE, which cleaves endothelial cytoskeleton, as well as E-cadherin and VE-cadherin that increase the permeability of the alveolar-capillary barrier [90]. Other NET-derived components such as cathespin G can degrade anti-inflammatory proteins via pro-inflammatory protein production, LL-37 promotes apoptosis in epithelial and endothelial cells and ROS produced by MPO causes both apoptosis and necrosis in epithelial cells [90]. Moreover, extracellular histones (H3 and H4) released from NETs are implicated as pivotal effectors of C5aR- and C5L2-mediated (C5a receptors) ALI in humans, rats and mice [91]. NETs are also found in models of sterile injury such as transfusion-related ALI (TRALI) [92]. NETs have been linked to ALI in influenza pneumonitis where NETs caused lung injury via association with alveoli in areas of tissue injury [93]. NETs are found in the sputum of CF patients [94]. The majority of extracellular DNA found in the sputum of CF patients is in fact NET derived, as the DNA complexes are consistent with neutrophil ETosis and share a similar protein signature [95]. Extracellular DNA leads to an increase in sputum viscosity that correlates with a high concentration of neutrophils and NET accumulation in CF airways that consequently aids microbial colonisation, proliferation and biofilm formation causing chronic inflammation correlating with increased pulmonary obstruction [96, 97]. Yet, why more ETosis is occurring in CF airways remains unclear. However, it is likely that NETs are formed in response to host bacteria, such as opportunistic *Pseudomonas aeruginosa*, one of the main pathogens to colonise the CF lung which is also a common pathogen known to induce NETs [97]. Both EETs and NETs are found in the airways of human atopic asthma patients in vivo [98], whereas NETs decorated with NE, histone H1 and citrullinated histone H3 are found in sputum of COPD patients [99, 100]. Interestingly, both *Mycobacterium* genotypes *M. tuberculosis* (cause of most types of tuberculosis) and *M. canetti* induced NET formation and ROS production in a time-dependent manner [101]. *M. tuberculosis-*induced NETs were decorated with key ETotic markers such as histone H2A, H2B and NE and were able to trap but not kill *M. tuberculosis* [101]. Granulomas are an important and hallmark feature of tuberculosis and are generally caused by mycobacterial or fungal infections. These prominent structures represent a key immune response to foreign material that is too large to be cleared by other immune defence processes. For an in-depth review of the role of ETosis during lung inflammation, refer to Cheng and Palaniyar [102]. Interestingly, there appears to be a link between NADPH oxidase activation, ETosis and apoptosis in immune defence against infectious agents. This has been highlighted by studies involving neutrophils obtained from patients with chronic granulomatous disease (CGD; a rare inherited disorder of NADPH oxidase) and mouse models of CGD, where in both instances, the ETotic response is severely diminished [76, 103]. Furthermore, following phagocytosis (in vitro), neutrophil apoptosis is compromised in CGD sufferers [104]. Failed resolution of inflammation in patients with CGD can lead to a number of inflammatory lung conditions including pneumonia, pulmonary fibrosis and lung abscesses, and specifically, in CGD mice, ALI can result as a consequence of impaired tryptophan catabolism (a superoxide-dependent process) [105].

Additional cell death processes play important roles during lung inflammation; these include autophagy and necroptosis. Autophagy entails the intracellular degradation of cellular components, which are then delivered to the lysosome for enzymatic degradation. Autophagy can play opposing roles during chronic lung inflammatory disorders and lung cancer. An increase in autophagy markers, such as autophagosome formation, and levels of LC3B-II (autophagosome-associated protein) are found in the pulmonary epithelium after induction of ALI in mice after extended exposure to hyperoxia [106]. During tuberculosis, autophagy can assist in the generation of anti-virulence factors [107], whereas during influenza A, infection autophagy is induced with viral replication dependent upon autophagosome formation [108]. Mitophagy (selective degradation of mitochondria via autophagy) can, in certain instances, aggravate the severity of COPD by activating additional cell death processes, whereas during pulmonary hypertension, autophagy can regulate cell death facilitating host defence [106]. Furthermore, autophagic degradation and clearance of cilia (ciliophagy) result in COPD-associated cilium dysfunction [109]. Impairment of autophagy can escalate the severity of cystic fibrosis and idiopathic pulmonary fibrosis, and in lung cancer, it can reduce carcinogenesis; yet it can also promote tumour cell survival. Therefore, autophagy can control the effectiveness of certain cancer therapies [106]. Conversely, necroptosis (programmed necrosis) is known to augment lung inflammation in several murine models. In a model of erythrocyte transfusion and LPS-induced lung inflammation, necroptosis of lung endothelial cells is induced via high mobility group box 1 (HMGB1) protein [110]. *Staphylococcus aureus* toxins can induce necroptosis via receptor-interacting protein kinases (RIP) 1 and 2 which bind to pro-necrotic mixed lineage kinase domain-like (MLKL) protein via RIP1/RIP2/MLKL signalling, which results in depletion of alveolar macrophages as well as IL-1β expression leading to pulmonary damage [111]. Necroptosis was also observed in bronchial epithelial cells in vitro via induction by cigarette smoke, which also triggered the release of DAMPs and pro-inflammatory cytokines (IL-8, IL-6) [112]. In vivo, cigarette smoke caused neutrophilic airway inflammation as evidenced by increased the number of neutrophils present in the BAL fluid, which was significantly reduced by treatment with the necroptosis inhibitor, necrostatin-1 [112].

### Efferocytosis

A critical process in the successful resolution on inflammation is the efficient clearance of apoptotic cells by phagocytes during a process termed efferocytosis. This process helps to limit inflammation and maintain tissue homeostasis. Efferocytosis ensures the swift removal of apoptotic cells before they lose membrane integrity and release their histotoxic intracellular contents to surrounding tissues, which would cause host tissue damage and exacerbate inflammation. Apoptotic cells once engulfed are contained within a large fluid-filed vesicle termed an efferosome that fuses with lysosomes to form the efferolysosome, which eventually digests the redundant cell. Efferocytosis is typically performed by professional phagocytes such as macrophages or dendritic cells; however, this process can also be performed by non-professional phagocytes such as epithelial cells and fibroblasts. Before phagocytosis, apoptotic cells undergo characteristic morphological changes such as cell shrinkage, membrane blebbing and karyorrhexis, which enable the dying cell to be recognised, engulfed and subsequently cleared. In most cases, phagocytes engulf dying cells in their entirety, such is the case for macrophages for the clearance of apoptotic neutrophils [113]. However, in certain instances, such as when the target is too large to be efficiently phagocytosed, multiple phagocytes can work in concert by engulfing apoptotic cells in ‘bite-sized’ portions. Such a scenario is observed during efferocytosis performed by fibroblasts in the absence of macrophages [114] .

#### Mechanisms

Recently reviewed by Poon et al., some of the key mechanisms of the efferocytosis process include the release of various find-me and ‘keep-out’ signals, as well as the presentation of various eat-me and ‘do not eat-me’ signals by apoptotic cells [10]. During early apoptosis, dying cells attract phagocytes via the release of chemotactic factors. These find-me signals can either be soluble or signal through submicron membrane vesicles. Soluble factors include nucleotides that are released from apoptotic cells through caspase-activated pannexin 1 (PANX1) membrane channels [10]. Signalling through submicron membrane vesicles includes microparticle-associated molecules such as CX3 chemokine ligand 1 (CX_3_CL1), ICAM3 and the Ca^2+^-dependent phospholipid-binding protein annexin A1 [10]. Annexin A1 is released when membrane integrity is lost during late apoptosis (secondary necrosis) and is known to promote recruitment of monocytes via proteolytic processing of a dis-integrin and metalloproteinase domain-containing protein 10 (ADAM10) [115] as well as promoting apoptotic cell engulfment and clearance [115, 116]. Interestingly, apoptotic cells also possess the ability to deter recruitment of pro-inflammatory cells via release of keep-out signals, which function as negative regulators of granulocyte migration. At present, the glycoprotein lactoferrin is the only known keep-out signal and is released from various apoptotic cell types, which subsequently inhibits neutrophil migration in vitro and in vivo [117] and eosinophil migration in vitro [118]. Various cell surface eat-me signals help phagocytes distinguish viable cells from apoptotic cells. The main eat-me signal exposed on the surface of apoptotic cells is the membrane phospholipid PS [66]. In viable cells, PS is confined to the inner membrane via the transmembrane lipid transporter protein flippase. However, during early-stage apoptosis, PS is translocated from the inner to the outer membrane leaflet via the activity of phospholipid scramblase. PS exposed on the surface of apoptotic cells can be detected by phagocytes via several recognition mechanisms. Direct detection of PS occurs via different membrane receptors, including brain-specific angiogenesis inhibitor 1 (BAI1) [119], stabilin-2 [120] and protein family members of the T cell immunoglobulin domain (TIM), specifically TIM1, TIM3 and TIM4 [121, 122]. Recognition of PS via BAI1 results in rearrangement of the cytoskeleton to aid phagocytic engulfment, which is mediated via the engulfment and cell motility protein 1 (ELMO1)-dedicator of cytokinesis-180 (DOCK180)-Ras-related C3 *botulinum* toxin substrate (RAC) (ELMO1-DOCK180-RAC) complex [119]. Stabilin-2 initiates apoptotic cell uptake via PS binding mediated by interactions with the engulfment adapter protein (GULP) and thymosin β4 (regulates actin polymerisation) [123, 124], whereas TIM4 predominantly functions as a tethering protein for PS where phagocytic engulfment is facilitated via signalling of associated proteins [125]. Aside from these genuine PS membrane receptors, PS can also be bound by bridging molecules including milk fat globule-endothelial growth factor 8 (MFG-E8), protein S and Gas6 which are ligands recognised by their cell surface receptors on phagocytes, specifically the α_v_β_3_ integrin family of receptors in the case of MFG-E8 and the Tyro3-Axl-Mer (TAM) family of receptors in the case of protein S and Gas6 [126–128]. Apoptotic cells can also expose calreticulin (endoplasmic reticulum resident protein 60) on their surface, which can serve as an additional eat-me signal. For example, translocation of calreticulin from the endoplasmic reticulum to the plasma membrane occurs during induction of apoptosis accompanied by endoplasmic reticulum stress in cancer cells, which are in turn subsequently cleared by phagocytes via CD91 (low-density lipoprotein-receptor protein) detection of calreticulin [129, 130]. In contrast to eat-me signals, cells can also expose do not eat-me signals on their cell surface. This is the case for viable cells that can, under certain physiological circumstances, translocate PS to their outer membrane leaflet. However, these viable cells avoid phagocytic uptake by exposure of do not eat-me signals such as CD31, CD47 and CD46 [10].

#### Regulation

Phagocytic functions can be augmented by exposure or treatment with glucocorticoids (GCs). GCs can also stimulate macrophages to switch to an anti-inflammatory phenotype (M2) where they shut down release of pro-inflammatory cytokines and simultaneously release anti-inflammatory cytokines (IL-10, TGFβ, IL-1ra), helping to promote resolution of inflammation and tissue repair. GCs are a class of corticosteroids which are a class of steroid hormones regularly used for the treatment of inflammatory diseases due to their potent anti-inflammatory properties. Cortisol is an important endogenous GC heavily involved in modulation of various metabolic, homeostatic, immunologic and cardiovascular functions. However, under certain chronic inflammatory conditions, endogenous levels of cortisol are not enough to suppress such inflammatory insults. In such instances, synthetic (exogenous) GCs such as dexamethasone and hydrocortisone (which can be more potent than endogenous GC counterparts) can be administered to aid and accelerate the resolution phase of inflammation. With regard to lung inflammation, GCs are commonly used limit inflammation during lung diseases such as asthma and ALI/ARDS.

Macrophage efferocytosis of neutrophils is enhanced in the presence of GCs such as dexamethasone and hydrocortisone in vitro [131]. Dexamethasone-treated macrophages also display structural reorganisation of the cytoskeleton and an increase in cell motility, both essential for efficient phagocytosis [132]. Furthermore, dexamethasone augmented the expression of active RAC in macrophages, a key signalling protein involved in a variety of cellular functions, including phagocytosis as well as cell motility, mitosis and wound healing [132]. GCs induce protein S-dependent efferocytosis through Mer receptor tyrosine kinase signalling, a member of the TAM receptor tyrosine kinase family [133, 134]. TAMs initiate signals that regulate cellular function as well as dictating the binding capacity and phagocytic clearance of apoptotic cells. TAM-deficient mice exhibit impaired efferocytosis capabilities, which are associated with several autoimmune diseases. At site of inflammation, GCs can stimulate Mer expression on phagocytes [131]. Inhibition of Mer-mediated efferocytosis in mice exacerbated LPS-induced lung injury, which was reduced by Mer-signalling upregulation via TNFα protease inhibitor-0 (TAPI-0), a specific inhibitor of Mer cleavage [135]. This highlights that Mer-mediated efferocytosis is a critical process which can modulate lung pathophysiology. It has been established that proteolytic cleavage from the cell membrane of phagocytes can downregulate Mer after exposure to pro-inflammatory stimuli such as LPS and bleomycin, with inhibition of this proteolytic cleavage successfully achieved via TAPI-0 blockade, which subsequently inhibits downregulation of Mer and augments efferocytosis in mouse models of LPS and bleomycin-induce lung injury [136, 137]. Deciphering the molecular mechanisms underpinning efferocytosis and its regulation via interaction with GCs will help facilitate the identification of novel therapeutic targets to promote the resolution of inflammation and tissue repair in the lung, as well as other organs. It is important to note that the efficacy of GCs during resolution if inflammation is dependent upon environmental milieu. In vitro GCs can stimulate eosinophil apoptosis; however, they are also known to delay neutrophil apoptosis. Yet during hypoxia, the GC-induced and pro-inflammatory cytokine-induced pro-survival effects upon neutrophil survival are lost [138].

Another way GCs are thought to exert and modulate their anti-inflammatory capabilities is via the expression and function of the 37-kDa protein annexin A1 (also known as lipocortin 1), a downstream effector molecule [139]. Annexin A1 signals through a G-protein coupled receptor (GPCR) known as formyl peptide receptor 2 (FPR2; ALXR in humans), which is also the receptor for the bioactive pro-resolving lipid lipoxin A_4_ [140]. Annexin A1 binds to acidic membrane phospholipids in a Ca^2+^-dependent manner and is expressed in high levels in the cytoplasm of resting cells. In human neutrophils, >60 % of cytoplasmic annexin A1 is stored in gelatinase granules [141]. Following cell activation (e.g. in response to inflammatory stimuli), rapid translocation of annexin A1 to the outer membrane leaflet takes place, where this find-me signal is then secreted via different cell-specific molecular mechanisms [139]. Endogenous annexin A1 liberation from apoptotic neutrophils and GC (dexamethasone)-treated macrophages enhances macrophage efferocytosis of neutrophils in vitro [142, 143]. Levels of annexin A1 expression from circulating neutrophils and monocytes are increased in healthy volunteers after GC administration [144], with expression levels of annexin A1 also modulated during disease. In Cushing’s disease (associated with elevated levels of cortisol), leukocytes exhibit markedly raised levels of intracellular annexin A1, and in Addison’s disease (associated with reduced levels of cortisol), leukocytes exhibit markedly lower levels of intracellular annexin A1 compared to healthy controls [145]. Innate immune cell release of annexin A1 following GC treatment can stimulate neutrophil apoptosis and macrophage efferocytosis and inhibit neutrophil transendothelial migration [139]. In vivo data from a mouse model of acute inflammation highlight annexin A1 as a key regulator during natural and GC-induced resolution of inflammation [146]. Nonetheless, the mechanism of GC regulation of annexin A1 remains largely unclear. Incidentally, efferocytosis can also be augmented in vivo in the murine lung and in alveolar macrophages from COPD patients by treatment with statins (cholesterol-lowering drugs), specifically by treatment with lovastatin which enhances efferocytosis via RhoA inhibition (cytoskeleton regulator) [147].

### Successful resolution of lung inflammation

Efferocytosis performed by resident lung phagocytes govern the successful resolution of lung inflammation and regulate normal lung structure. Professional phagocytes include alveolar macrophages, interstitial lung macrophages and lung dendritic cells, whereas non-professional phagocytes include lung epithelial cells such as alveolar and bronchial epithelial cells. Defective efferocytosis which results in an increased number of apoptotic cells is implicated in a number of lung diseases including asthma, ALI, CF and COPD and is well reviewed by [148, 149]. Furthermore, highly specialised bioactive lipids play key roles during the resolution phase of inflammation.

#### Professional lung phagocytes

Alveolar macrophages comprise the most abundant population of professional phagocytes within the alveolar space where they can make up 90–95 % of the cell population from healthy BAL fluid. These phagocytes possess vast phagolysosomal capacity which serves to kill ingested microbes. Apoptosis of alveolar macrophages which have ingested *Streptococcus pneumonia* is essential for the killing and clearance of these bacteria, which contribute to resolution in a mouse model of pulmonary infection [150, 151]. Although alveolar macrophages are capable of phagocytosing a diverse array of injurious agents, at rest, rates of alveolar macrophage efferocytosis are somewhat lower than those of other tissue resident macrophages [152]. Defects in alveolar macrophage efferocytosis are attributable to several factors including diminished adhesion and via SP-A and SP-D (efficient regulators of macrophage function) activation of transmembrane receptor signal inhibitory regulatory protein alpha (SIRPα) [153]. However, these efferocytosis defects are overcome via recruitment of mononuclear phagocytes during acute inflammatory scenarios such as acute pulmonary inflammation [153]. In contrast to circulating monocytes, dendritic cells and tissue resident macrophages, alveolar macrophages possess numerous apoptotic cell recognition receptors, which imply that these cells are extremely responsive to apoptotic cell death in the alveolar space [154]. Alveolar macrophages highly express all three TAM receptors, with blockade of these receptors shown to further suppress efferocytosis but not phagocytic binding [152]. The lungs can also play host to elevated numbers of interstitial macrophages, commonly observed in smokers and COPD patients. Although the efferocytosis capabilities of interstitial lung macrophages remain largely unclear, they appear to play a key role in promoting the pathogenesis of cigarette smoke-induced emphysema in mice via TNF and IL-6 release [155]. Within the lung, there are two dendritic cell subsets, and it has been demonstrated, in the murine lung, that the CD103^+^ dendritic cell subset facilitates efferocytosis and presents apoptotic cell-associated antigens to CD8^+^ T cells [156]. Similar to efferocytosis capabilities of interstitial lung macrophages, there is also a paucity of investigations upon the efferocytosis capabilities of human lung dendritic cells, which is an area that also deserves more attention.

#### Non-professional lung phagocytes

Recognition and subsequent efferocytosis of eosinophils by human bronchial and alveolar epithelial cells have been reported in vitro, which were augmented by dexamethasone treatment [157, 158]. Recognition of apoptotic eosinophils was found to be both lectin- and integrin-dependent [157] with apoptotic cell engulfment involving αvβ3-, CD36-, αvβ5- and PS receptor-mediated events [158]. These findings therefore imply a non-passive role for the airway epithelium during eosinophilic-dominant inflammation in asthma. More recently, in transgenic mouse models of allergic airway inflammation, the Ravichandran group were able to demonstrate RAC1-dependent efferocytosis of airway epithelial cells by bronchial epithelial cells, which resulted in liberation of anti-inflammatory cytokines [159] (see Table 1 for an overview of anti-inflammatory cytokines and associated bioactivities). Incidentally, inducible deletion of RAC1 expression in mouse airway epithelial cells resulted in impaired efferocytosis and generation of an atypical anti-inflammatory cytokine profile in bronchial epithelial cells [159]. Yet, of present, there remains a distinct lack of investigations attempting to manipulate efferocytosis capabilities in lung epithelial cells in current models of airway inflammation. Facilitation of such investigations could help unearth novel therapeutic approaches for the treatment of various inflammatory lung diseases.

**Table 1:** Anti-inflammatory bioactivities of various cytokines

Cytokine	Main source(s)	Major anti-inflammatory bioactivities
IL-1ra	Monocytes/macrophages, T cells, B cells and dendritic cells	Specifically inhibits the activity of pro-inflammatory IL-1α and IL-1β
IL-2	TH cells	Modulates cellular and humoral responses during chronic inflammation, increases T cell proliferation, lymphokine secretion and augments expression of MHC class II molecules
IL-4	T cells (TH2), B cells, mast cells and basophils	Inhibits generation of monocyte-derived pro-inflammatory cytokines IL-1, IL-6, IL-8, TNF and MIP-1α; decreases macrophage cytotoxic activity and NO production; stimulated IL-1ra synthesis; augments MHC class II expression on B cells; and promotes B cell, T cell and mast cell development
IL-6	T cells, B cells, neutrophils, monocytes/macrophages, PMN leukocytes and fibroblasts	Inhibits pro-inflammatory TNF, IL-1, GM-CSF, IFNγ and MIP-2 generation and stimulates synthesis of glucocorticoids and IL-1ra
IL-10	Monocytes/macrophages, T cells (TH2) and B cells	Inhibits generation of monocyte/macrophage-derived pro-inflammatory TNF, GM-CSF, MIP-1α, MIP-2α, IL-1, IL-6, IL-8 and IL-12 and also attenuates pro-inflammatory cytokine generation in neutrophils and mast cells
IL-11	Stromal cells, fibroblasts, epithelial cells and osteoblasts	Inhibits generation of pro-inflammatory IL-1 and TNF generation from macrophages and stimulates TH2 cell responses
IL-13	T cells (TH2)	Inhibits generation of pro-inflammatory IL-1β, IL-6, IL-8 and TNF generation by monocytes and augments differentiation and proliferation of monocytes and B cells
IL-22	T cells, NK cells and dendritic cells,	Induces proliferative and anti-apoptotic pathways and production of AMPs which serve to block tissue destruction and support tissue repair and modulates tissue responses during intestinal inflammation
IL-27	T cells, monocytes, neutrophils, NK cells, mast cells and bronchial epithelial cells	Regulates T cell responses and differentiation and limits pro-inflammatory cytokine production
IL-35	Regulatory B and T cells	Stimulates T cell proliferation and anti-inflammatory IL-10 and TGFβ generation
IL-37	Macrophages and epithelial cells	Decreases generation of pro-inflammatory cytokines
IL-38	PBMCs	Decreases generation of IL-8 and T cell cytokines
TGFβ	T cells, monocytes/macrophages, neutrophils, platelets, alveolar epithelial/endothelial cells and fibroblasts	Inhibits leukocyte adhesion and monocyte/macrophage pro-inflammatory cytokine generation and promotes wound healing/angiogenesis
IFNα	Monocytes/macrophages, PMN leukocytes, plasmacytoid dendritic cells, alveolar epithelial cells and fibroblasts	Induces anti-inflammatory IL-1ra and IL-10 generation and inhibits pro-inflammatory IL-1, IL-8 and TNF generation

*IL-1ra* interleukin-1 receptor agonist, *TH* T helper, *MHC* major histocompatibility complex, *MIP-1α* macrophage inflammatory protein-1 alpha, *NO* nitric oxide, *PMN* polymorphonucleated, *AMPs* anti-microbial peptides, *PBMCs* peripheral blood mononucleated cells

#### Pro-resolving bioactive lipids

Lipoxygenase (LOX)-dependent enzymatic conversion of polyunsaturated fatty acid (PUFA) substrates to PUFA-derived mediators is another important biochemical process which is key to modulating inflammation [5, 160]. PUFAs are released from the cell membranes of activated cells such as neutrophils, monocytes/macrophages, lymphocytes and eosinophils, then are enzymatically converted to specialised pro-resolution lipid mediators, which vary in structure and function [161]. These pro-resolving lipid mediators include lipoxins (A_4_ and B_4_), D- and E-series resolvins, protectins and maresins and are active from picogram to nanogram scales. These bioactive lipids often possess dual anti-inflammatory and pro-resolution bioactivities (see Table 2 for overview), which are excellently reviewed by the Serhan group [21, 162, 163]. In clinic, pharmacological inhibitors including those that target certain lipoxygenases are used due to their ability to suppress adverse events which accompany inflammation; however, these inhibitors can also impair endogenous production of other bioactive lipids [164–166]. Contrastingly, aspirin and the glucocorticoid dexamethasone can initiate endogenous anti-inflammatory pathways via activation of the lipoxin A_4_ receptor AXLR/formyl peptide receptor like-1 receptor (FPRL1) [140], a GPCR which is now termed AXLR/FPR2 [167]. These pro-resolving bioactive lipids are implicated in a number of inflammatory lung diseases, including asthma, cystic fibrosis, interstitial lung disease, aspirin-exacerbated respiratory disease, COPD and emphysema [161].

**Table 2:** Specialised bioactive lipids which promote the resolution of inflammation

Bioactive lipid	Main source	Major anti-inflammatory and pro-resolution bioactivities
Lipoxins	ADA	
Lipoxin A_4_		Neutrophils reduce chemotaxis/recruitment/transendothelial/epithelial migration, epithelial cell interactions, number of apoptotic neutrophils, O2^−^ generation and degranulation
		Monocytes stimulate chemotaxis/adhesion and reduce peroxynitrite generation
		Macrophages enhanced efferocytosis of neutrophils
		Eosinophils reduce migration/chemotaxis and generation of IL-5 and eotaxin
		NK cells reduce cytotoxicity and increase granulocyte apoptosis
		Dendritic cells reduce generation of IL-12
		Epithelial cells reduce the release of IL-6 and IL-8
		Endothelial cells reduce ROS generation and VEGF-induced migration
		Other decreases vascular leakage and adherent capabilities of leukocytes
Lipoxin B_4_		Leukocytes modulate the adherence and motility of neutrophils/monocytes and inhibit neutrophil infiltration and stimulate macrophage recruitment
D-resolvins	DHA	
Resolvin D1		Neutrophils reduce recruitment and transmigration
		Macrophages stimulate/augment phagocytosis of apoptotic cells and allergens, induce M2 macrophage phenotype and reduce LPS-induced TNF release
		Other reduces resolution interval, oxidative stress, pro-inflammatory cytokines in BAL fluid and levels of prostaglandins/leukotrienes and augment microbial clearance
Resolvin D2		Leukocytes reduce neutrophil infiltration and leukocyte-endothelial cell interactions
Resolvin D3		Leukocytes reduce neutrophil transmigration and augment macrophage phagocytosis/efferocytosis
Resolvin D4		Leukocytes reduce neutrophil infiltration and augment macrophage efferocytosis of neutrophils and phagocytic clearance of *Staphylococcus aureus*
		Other augments fibroblast efferocytosis of neutrophils
E-resolvins	EPA	
Resolvin E1		Neutrophils reduce O2^−^ generation and transendothelial/epithelial migration
		Monocytes decrease cell number
		Macrophages augment efferocytosis of neutrophils
		Eosinophils/lymphocytes reduce recruitment
		Dendritic cells reduce migration and IL-12 generation
		Other modulates the production of chemokines/cytokines and stimulates anti-apoptotic signals and reparative processes in inflamed tissues
Resolvin E2		Leukocytes modulate neutrophil chemotaxis, augment phagocytosis and generation of anti-inflammatory cytokines, efficiently downregulate the surface expression of integrins and reduce responses to PAF
Resolvin E3		Neutrophils reduce infiltration
Protectins	DHA	
Protectin D1		Neutrophils reduce infiltration, transmigration and TNF/IFNγ generation
		Macrophages modulate function and stimulate efferocytosis of PMN leukocytes
		Other modulates chemokine/cytokine production and migration of T cells and reduces eosinophil chemotaxis/adhesion
Maresins	DHA	
Maresin 1		Neutrophils reduce numbers in peritonitis exudates
		Macrophages augment phagocytic capabilities
		Other reduces PMN leukocyte transendothelial cell migration and dust-induced cytokine production in bronchial epithelial cells and aids tissue regeneration

*Source*: [193, 194]

*ADA* arachidonic acid, *O2* ^*−*^ superoxide anion radical, *VEGF* vascular endothelial growth factor, *EPA* eicosapentaenoic acid, *DHA* docosahexaenoic acid, *BAL* bronchial alveolar lavage, *PAF* platelet-activating factor, *PMN* polymorphonucleated

#### Lipoxins and resolvins

At sites of inflammation, the PUFA arachidonic acid (ADA) can be metabolised to prostaglandins and leukotrienes (such as LTB_4_), but it is also converted to a family of pro-resolving bioactive lipids termed lipoxins, which can suppress leukotriene-induced inflammation [168]. Presently, lipoxins are the most studied family of pro-resolution lipids and their levels considerably increase during the resolution phase of inflammation [5]. During airway inflammation, enzymatic hydrolysis via neutrophil 5-LOX and epithelial 15-LOX activity leads to lipoxin A_4_ and B_4_ biosynthesis [4]. Additional lipoxin A_4_ and B_4_ biosynthesis can occur via platelet and neutrophil interactions (via platelet 12–LOX) such as in the vasculature [169] as well as by various cell types including neutrophils, eosinophils and alveolar macrophages, albeit to a lesser extent [5, 169, 170]. As well as ADA, additional PUFAs are present at sites of inflammation such as docosahexaenoic acid (DHA) and eicosapentaenoic acid (EPA), which can be enzymatically converted to D- and E-series resolvins, respectively. During vascular inflammation, DHA is converted by aspirin-acetylated endothelial cell-derived cyclooxygenase-2 (COX-2) via neutrophil LOX activity, which leads to hydrolysis of D-series resolvins [161]. In a similar inflammatory scenario, EPA is converted to E-series resolvins by aspirin-acetylated endothelial cell-derived COX-2, where biosynthesis of E-series resolvins involves direct transformation of unstable intermediates of EPA by activated leukocytes [161]. The GPCR AXLR/FPR2 serves as the receptor for lipoxin A_4_ and resolvin D1 [171, 172], which can also be activated by annexin A1 via glucocorticoid induction [140]. Humans express ALXR/FPR2 in leukocytes and tissue resident cells [171] with receptor expression modulated by local inflammatory mediators. Resolvin D1 also binds to another GPCR, namely, GPR32, which is also known as the resolvin D1 receptor (DRV1) and expressed by leukocytes [173]. However, due to lack of GPR32 mobilisation from human neutrophils, the pro-resolution activities of resolvin D1 are brought about primarily by ALXR/FPR2 signalling pathways, which have confirmed that mice are lacking the ALXR/FPR2 receptor [174]. Resolvin E1 can bind to additional GPCRs, namely, the chemokine receptor-like 1 (CMKLR1) receptor and LTB_4_ receptor 1 (BLT1) expressed by polymorphonucleated (PMN) leukocytes [175].

#### Protectins and maresins

Protectins are generated via a 15-LOX-dependent manner which catalyses the conversion of DHA to protectin D1 via an epoxide intermediate [176]. Additionally, aspirin can initiate protectin biosynthesis from DHA via COX-2 acetylation [177]. The pro-resolving bioactions of protectin D1 are known to be cell-specific; therefore, it is likely that such interactions are mediated by one or more specific receptors. However, protectin 1-specific receptors remain to be identified [161]. Somewhat more recently, maresins (derived from macrophages) have been discovered, which are another family of pro-resolving bioactive lipids [178]. Akin to protectins and D-series resolvins, maresins are also derived from DHA. However, biosynthesis of maresin 1 occurs via a novel epoxide intermediate, which also enhances the conversion of macrophages from an M1 to M2 phenotype, with M2 macrophages able to produce elevated levels of this pro-resolving lipid mediator from the epoxide intermediate compared to M1 macrophages [179]. Importantly, in terms of pro-resolution, maresin 1 is also able to increase macrophage efferocytosis and aid tissue regeneration [178, 180]. Identification of maresin 1-specific receptors is yet to transpire; however, it is apparent that specific GPCRs are involved [180].

#### Bioactive lipids in lung inflammation

Impaired generation of these pro-resolving lipid mediators during airway inflammation can lead to chronic inflammatory lung diseases. Decreased lipoxin formation has been described in severe/uncontrolled asthma, cystic fibrosis, aspirin-exacerbated respiratory disease and scleroderma-interstitial lung disease [181–184]. During severe/uncontrolled asthma, dysregulated expression of lipoxin biosynthetic genes is partly responsible for the decreased production of lipoxins (see review by Levy et al. [185]). Elevated levels of DHA are found in the airway mucosa of heathy individuals; however, during asthma and cystic fibrosis, mucosal levels of DHA are reduced [186]. Resolvins E1 and D1 can enhance the resolution of allergic airway inflammation in mouse models of asthma [187, 188]. Resolvin E1 reduced the numbers of macrophages, eosinophils and lymphocytes present in BAL fluid as well as improving airway hyperreactivity and airway mucus metaplasia postinhalation of methacholine in mice [189]. Specifically, resolvin E1 enhances the resolution of allergic airway inflammation via reduction in IL-6, IL-17 and IL-23 production in the murine lung, with pro-resolution assisted via increased generation of IFNγ and lipoxin A_4_ in the lungs of mice treated with resolvin E1 [189]. Recently, resolvin D4 has been identified in human tissues and confirmed to have potent pro-resolving activities during murine *S. aureus* infections [190, 191]. However, the precise role of resolvin D4 during lung inflammation remains to be established. Akin to lipoxin levels during severe/uncontrolled asthma, a reduction in protectin D1 levels is also observed during acute asthma exacerbations [192]. Protectin D1 can also reduce allergic airway responses in a mouse model of asthma where intravenous administration of protectin D1 (prior to aeroallergen challenge) results in attenuation of eosinophil infiltration and pro-inflammatory cytokine release [192]. Furthermore, postallergen challenge (once pulmonary inflammation has been established), protectin D1 is able to exert pro-resolution properties which accelerate the resolution of allergic airway inflammation [192]. Taken together, the above evidence indicates that these bioactive lipids are key effectors of pro-resolution circuits during lung inflammation and that impairment in their endogenous levels contributes to several inflammatory lung diseases. For thorough review of the roles played by pro-resolution bioactive lipids during lung inflammation, refer to [161].

### Dysregulated/impaired resolution of lung inflammation

#### Neutrophil-dominant inflammation

As the most abundant cells, and in many ways the bluntest instruments of the immune armoury, it is perhaps unsurprising that neutrophilic inflammation is a hallmark of numerous inflammatory lung conditions. Furthermore, the lungs are prime sites for inflammation and injury as neutrophils persist in the lung far longer than other organs [195]. The first responders to both endogenous and exogenous stimuli, their role in acute disorders, are without question, yet they are also implicated in the pathogenesis of numerous chronic conditions, suggesting a failure of the normal mechanisms by which resolution proceeds. Below, we use several demonstrative conditions to illustrate the mechanisms of neutrophilic inflammation, resolution and development of chronicity.

#### Pneumonia

As a significant burden of morbidity and mortality both within the UK and the wider world, pneumonia is the acute inflammatory response to infection of the lower respiratory tract that is visible on a chest X-ray. It occurs in response to a variety of pathogens, most commonly bacteria and viruses, and is usually triggered by recognition of conserved ‘foreign’ receptors. Where the host response is successful in containing and engulfing the responsible pathogen, infection remains localised and the lung may heal without sequelae. Acute failure of this process results in disseminated infection, which may ultimately lead to death. In the intermediate to long term, failure of successful sequestration and resolution predisposes to empyema, abscess formation and bronchiectasis. Despite better understanding of the underlying mechanisms of inflammation and injury, the mainstay of treatment for pneumonia remains anti-microbials. Unfortunately, there remain a subset of patients in whom even a combination of host defence and anti-biotics fail to control infection and who go on to develop multi-organ failure (see below). In recent years, there has been more interest in exploring the role of modulating the immune response in severe pneumonia, and numerous different drugs have shown some potentials in improving outcomes.

*Streptococcus pneumoniae* is the most commonly implicated pathogen in community-acquired pneumonia (CAP) and can result in a spectrum of disease severity. Streptococcal infection is associated with dense neutrophilic inflammation and activation of the coagulation cascade, via the thrombin receptor, proteinase-activated receptor 1 (PAR-1). Numerous PAR-1 antagonists have been developed, and animal models suggest that suppression of coagulation activation via this pathway can reduce neutrophil airway load, inflammatory cytokine production and alveolar leak without compromising bacterial clearance [196]. Furthermore, proof of principal research suggests that existing anti-platelet agents well established in the management of cardiovascular disease may yet improve outcomes in pneumonia by reducing activation of the coagulation cascade and thereby progression to ALI and ARDS [197]. Activated protein C (APC) is an endogenous anti-inflammatory and anti-coagulant chemokine that is implicated in the prevention of disseminated infection and source control. It has recently been highlighted that in animal models of streptococcal pneumonia, overexpression of APC reduces bacterial spread to other organs and neutrophilic inflammation at the primary infection site [198]. As well as exploration of the pathways involved in the acute inflammatory response, there has been growing interest in drugs with an established role in the management of chronic lung diseases, such as macrolides. Macrolides have long been in favour as they exhibit both anti-bacterial and anti-inflammatory properties and are useful in conditions where bacterial colonisation is a hallmark, including diffuse pan-bronchiolitis, cystic fibrosis and bronchiectasis. A recent pilot study highlighted a trend towards reduced circulating pro-inflammatory cytokine levels in patients with CAP treated with macrolides vs. those treated with other classes of anti-microbials, particularly at 5-7 days postinfection [199]. In the past decade, it has been increasingly recognised that as well as reducing lipid burden and modifying cardiovascular disease, HMG Co-A reductase inhibitors (statins) have hitherto unappreciated anti-inflammatory effects that are potentially harnessable for management of inflammatory disease. A recent trial suggested that atorvastatin reduced cough severity in stable bronchiectasis with associated increase in apoptotic neutrophils seen in sputum, and there is renewed interest in their role in modulating acute inflammatory conditions [200]. A recent systematic review examined a number of studies exploring the role of statins in CAP and concluded that they modulate neutrophil response, reduce circulating cytokine burden and potentially impact mortality [201].

Neutrophil longevity is a key determinant of the inflammatory response and is an obvious target in the search for novel anti-inflammatory agents. In the setting of infection, the challenge is the safe depletion of neutrophil number to a level that is able to ameliorate short- and long-term morbidities without compromising host defence and predisposing to systemic infection. There is evidence in numerous acute and chronic lung conditions, including CAP, that failure of timely neutrophil apoptosis contributes to morbidity [202]. Cyclin-dependent kinase inhibitors (CDKis) are a group of drugs that are being extensively researched for their ability to arrest cell cycle progression and induce apoptosis even in terminally differentiated cells such as neutrophils via downregulation of Mcl-1, and there are now several studies which demonstrated their potential as safe and effective modulators of inflammation [203–205].

#### ARDS

Multi-organ dysfunction syndrome (MODS) is a frequently fatal condition caused by an overwhelming inflammatory insult that results in a paradoxically unhelpful aggressive mucosal response. ARDS is the respiratory component of this disorder and results from a pathological reaction known as diffuse alveolar damage, which occurs secondary to rampant neutrophilic inflammation [206]. There are many causes of ARDS, including sepsis, shock, trauma and gastric aspiration [207, 208]. Progression to ARDS is a marker of severe sepsis and is associated with poor outcomes. Therapy for MODS remains largely supportive, but there is growing interest in the role of immune modulation as an adjunct to anti-biotic or other therapy to dampen the hyperactive immune response that ultimately leads to severe impairment of gas exchange and respiratory failure. Mortality in ARDS correlates with neutrophil number and levels of circulating pro-inflammatory cytokines, suggesting that harnessing the inflammatory response may be the key to improving outcomes [209, 210].

Despite ongoing controversy, glucocorticoids remain the best studied anti-inflammatory strategy in ARDS. There is some evidence to suggest that given early in disease course, intravenous steroids reduce requirement for mechanical ventilation, length of ITU stay and improve oxygenation, with a modest effect on mortality [211–214]. Such success is, however, only likely to outweigh potential complications in the setting of vigilant surveillance for nosocomial infection and eschewal of neuromuscular blockade due to the potential complications of steroid treatment. Furthermore, it has been suggested that if left to later time points, i.e. >14 days postonset, steroid administration may cause a paradoxical increase in mortality [211]. Consequently, glucocorticoid therapy remains to be proven as an effective therapy for ARDS and is not recommended for treatment unless it is known to be secondary to a steroid-sensitive insult. Early studies in animal models suggest that macrolide anti-biotics may demonstrate efficacy in management of ARDS [215, 216]. Although this is supported by an observational study that highlights a trend towards reduced mortality with macrolide treatment, the evidence remains insufficiently robust to support their use as a routine management option [217, 218]. Perhaps almost as enlightening as those therapies that have demonstrated promise are those which despite good biochemical rationale have failed to prove clinically efficacious. Despite the known role of cyclo-oxygenase-derived metabolites in sepsis and its sequelae, use of non-steroidals has historically been demonstrated not to lead to a reduction in sepsis-associated ARDS [219].

#### Cystic fibrosis

CF is the most common life-limiting autosomal recessive condition in Caucasians, with an incidence of 1 in 2500. Caused by loss of function mutations of cystic fibrosis transmembrane conductance regulator (CFTR), an epithelial chloride channel, it is a heterogenous multi-system inflammatory disorder of which the major clinical manifestations are severe, progressive bronchiectasis and exocrine pancreatic insufficiency. The classical pathophysiological explanation for CF lung disease is the ‘low-volume hypothesis’, whereby abnormal airway surface electrochemical gradients secondary to loss of CFTR result in increased uptake of water and extracellular cations [220]. The resultant dehydration of airway surface liquid promotes mucus hypersecretion, inhibits mucociliary clearance and disables cationic host defence peptides, leading to incessant cycles of sinopulmonary infections that eventually progress to chronic inflammation with airway remodelling. Once structural lung disease has developed in CF, it is rare for it to regress, and consequently, there is a desperate need to develop effective early management options to reduce long-term morbidity [221].

In recent years, there has been a move to explore the role of aberrant immune function in CF patients, as it is evident that there are non-CFTR determinants of (lung) disease severity and numerous hints of abnormal inflammatory responses. The unusual susceptibility of CF patients to ‘low-virulence’ pathogens, such as *P. aeruginosa* and *Burkholderia cepacia*, increased incidence of allergic airway disease, and growing evidence of a ‘CF-related enteropathy’ and systemic inflammation are to name but a few [222–224]. Despite decades of research, until recently, there has been very little progress in development of new treatment options for CF, although promising drugs directly restoring the CFTR function have recently become available for a small subset of patients [225]. For the remainder of the CF population, perhaps the best hope lies in the development of safe and effective anti-inflammatory agents that can be used synergistically with anti-biotic therapies to prevent the establishment of chronic inflammation and airway damage.

A number of groups have demonstrated intrinsic failure of the innate immune system in CF, and debate continues over whether this is a result of a hitherto unappreciated role of CFTR in neutrophil function or secondary to a chronic inflammatory environment in CF adults. Delayed neutrophil apoptosis, aberrant phagolysosomal destruction of *Pseudomonas* and excess IL-8 production have all been described in CF patients and offer an array of therapeutic targets [223, 226, 227]. As discussed above, there is much hope that CDKis can offer a novel approach to immune modulation in inflammatory disorders as their role in managing non-malignant disorders is explored. There is preliminary evidence that CDKis can correct delayed apoptosis in CF neutrophils, offering hope of correcting the inflammatory response and perhaps increasing the efficacy of antimicrobials in potentiating clearance of established airway pathogens [227]. Indeed, with development of novel water-soluble CDK inhibitors, the option of nebulised therapy remains open and may reduce systemic effects whilst concentrating efficacy. As well as a paradoxical failure to clear pathogens, the persistence of neutrophils in the CF airway results in excess PMN-derived proteases, which not only damage respiratory epithelia directly but also reduce the efficiency of phagocytic clearance [228, 229].

Conventional anti-inflammatory strategies have historically been trialled in CF patients but remain limited by significant adverse effects, and they remain out of favour in routine clinical practice [230, 231]. There has been little exploration of the role of ‘topical’ traditional anti-inflammatories such as nebulised non-steroidal anti-inflammatory drugs (NSAIDs), although this route may offer a safe and more effective opportunity to ameliorate lung inflammation whilst minimising systemic effects [232]. Inhaled corticosteroids are generally reserved for those patients with concurrent airway hyperreactivity as they demonstrate a more steroid-responsive airway inflammatory infiltrate. Several groups have demonstrated increased LTB_4_ levels in the CF airway, and there has been some success in using montelukast, a leukotriene receptor antagonist well established in the management of asthma, to reduce respiratory symptoms in CF patients [233, 234]. Perhaps unsurprisingly, given the prevalence of microbial colonisation, macrolides have a long history in CF lung disease. Their role is generally accepted to be related to both their anti-inflammatory properties as well as delayed anti-microbial effects, which reduce pathogen burden [235–238]. Unfortunately, there is recent evidence to suggest that long-term macrolide use is associated with the increasing incidence of multi-resistant atypical mycobacterial infections in CF patients, which may ultimately limit their use [239]. One of the problems with novel agent development is the protracted period from bench to bedside, and as such, there is great interest in exploring alternative uses for drugs with well-established safety and tolerability profiles. Statins, long favoured for their role in serum lipid modulation, are now known to exhibit broader anti-inflammatory properties, and the potential implications of this are currently being explored in a variety of conditions. IL-8 is known to be abundant in CF serum, and it has recently been demonstrated that fluvastatin is able to reduce IL-8 levels and may ultimately help to suppress systemic inflammation [240].

#### Eosinophil-dominant inflammation (asthma)

Eosinophils are important during allergic airway inflammation. The combined injurious effects resulting from high numbers of infiltrating eosinophils, delayed eosinophil apoptosis and impaired efferocytosis can cause chronic inflammatory lung disease, such as asthma. Asthma is a spectrum of conditions defined by the common pathology of reversible airway obstruction and hypersensitivity of the respiratory mucosa to environmental antigens. Most commonly, it occurs as part of an allergic syndrome of atopic disorders, though may occur in isolation. Asthma patients develop sensitivity to environmental antigens such as animal dander and plant material and on exposure to those antigens, develop a type 1 allergic response resulting in bronchoconstriction, wheeze, cough and mucus hypersecretion that lead to airflow limitation.

Affecting approximately 10 % of UK adults, asthma is a common disorder of multi-factorial origins, with research indicating that both genetics and the environment have a significant role to play. In predisposed individuals, ‘normal’ environmental antigens transmigrate through the airway epithelia and are presented to naïve T cells, which trigger activation of IgE production by B cells. IgE interacts with receptors on the surface of tissue resident mast cells, and further exposure to the antigen results in IgE cross-linkage with cell activation. The resultant mast cell degranulation causes release of mediators including histamine, LTB_4_, IL-8 and IL-10 and TNF, precipitating an acute inflammatory response. The late-phase asthmatic response classically occurs 6–9 h after antigen exposure and occurs secondary to the persistent secretion of cytokines, e.g. IL-5, GM-CSF and IL-3, which promote eosinophil migration, persistence and longevity in the lungs and form the bass of persistent airway inflammation in asthma patients.

The mainstay of asthma treatment is glucocorticoids, generally administered as inhaled preparations, which blunt the inflammatory response and trigger eosinophil apoptosis. This therapy, with adjunct bronchodilators, is sufficient to control symptoms in the majority of patients but lacks subtlety and is significantly limited by toxicity. Perhaps unsurprisingly, there remains a subset of patients who fail to respond to this approach and have persistently uncontrolled symptoms that may ultimately lead to airway remodelling. Consequently, there is a need for novel, specific inhibitors of eosinophilic inflammation in the lung, which can sufficiently control symptoms and display minimal systemic toxic effects [241]. There has been good success with the use of adjunct leukotriene receptor antagonists in the management of moderate-severe asthma, which selectively inhibit the pro-inflammatory effects of leukotrienes, highlighting that the principle of targeted therapies is sound [242].

Delayed eosinophil apoptosis and thereby persistence in the airway remains a core pathological feature of asthma and is one of the targets of steroid therapy. Recent in vitro and murine studies have demonstrated that as in neutrophils, CDK inhibitors are able to induce apoptosis of both circulating and inflammatory eosinophils via downregulation of Mcl-1, although the significance of this in the clinical setting remains unclear [243–245]. A recent study examining novel modulators of eosinophil apoptosis has highlighted that hydrogen peroxide induces cell death and accelerates resolution of airway inflammation in a caspase-dependent manner, as well as accelerating recovery of lung function [246]. As previously discussed, in recent years, there has been much emphasis on the role of endogenous lipid mediators of resolution, e.g. lipoxins, resolvins and protectins, and the potential role they may play in ameliorating the harmful response in inflammatory conditions [69]. Resolvin D1, one such mediator, and its counterpart aspirin-triggered resolvin D1 have been shown to significantly reduce airway eosinophilia and mucus hypersecretion via reduction of IL-5 degradation [188]. Lipoxin A_4_ has been reported to downregulate eosinophil responses via the suppression of activation by GM-CSF [247]. Its functionally related, though structurally distinct counterpart lipoxin B_4_, promotes resolution of allergic resolution in upper and lower airways via reduced eosinophil chemotaxis and mast cell degranulation, emphasising the potential for therapeutic harnessing of these pathways in eosinophilic airway disorders [248]. Flavones are a recently described group of polyphenolic compounds with potential anti-inflammatory and anti-malignant properties, which have been the subject of much research interest. The flavone wogonin has been shown to modulate granulocyte apoptosis via suppression of Mcl-1 and CDK-9, both in vivo and in vitro, therefore highlighting a potential role for flavones in atopic disorders [249–251].

## Conclusion

In the lung, prompt resolution of acute inflammatory responses occurs regularly, aiding to preserve a healthy state within the host. In most cases, this process is instigated by neutrophils, but in certain scenarios, eosinophils can dominate; where in either case, these granulocytes respond to noxious respiratory stimuli such as airborne pathogens, allergens and foreign particles. The outcome of acute inflammation in the lung is regulated by a balance between the presence of different sets of mediators and specific receptors. These mediators and receptors either serve to exacerbate the inflammatory response, which can lead to chronic lung inflammation and the onset of diseases such as ARDS, CF, COPD and asthma, or they can dampen inflammation and contribute to returning the lung to a healthy state via pro-resolution and pro-reparative processes. We have attempted to report the current understandings with regard to mechanisms central to controlling the resolution of lung inflammation and injury. Clearly, enhanced efferocytosis of apoptotic neutrophils/eosinophils performed by anti-inflammatory and/or pro-resolution macrophages, who in turn shut down their release of pro-inflammatory stimuli and increase their release of pro-resolution/reparative mediators, remains a key process for successful resolution of inflammation and repair. More recently, however, it has become evident that specialised bioactive lipid mediators belonging to the lipoxin, resolvin, protectin and maresin families can also modulate inflammation by contributing to the pro-resolution process. The biological actions of pro-resolving lipids are stereospecific, receptor-mediated and extremely potent even at picogram and nanogram concentrations [161]. Additionally, these pro-resolving lipids selectively interact with key cell types involved in innate immune defence, where they can have cell-type specific actions upon neutrophils, macrophages and endothelial cells [161]. In the lung, such actions include termination of leukocyte infiltration; return to normal vascular permeability via concomitant reduction in pulmonary edema, neutrophil apoptosis, non-inflammatory infiltration of monocytes/macrophages and macrophage efferocytosis of such neutrophils; and macrophage removal of respiratory pathogens and necrotic remnants. These actions all assist in the successful resolution of lung inflammation and injury to return the lungs to normal homeostasis and health. Further identification of mediators and the mechanisms by which they contribute to resolution of lung inflammation will help provide novel therapeutic strategies for the treatment of lung disease and injury.
